# International Brazilian Journal of Urology reaches the highest impact factor in its history (3,050) and changes level in this current management

**DOI:** 10.1590/S1677-5538.IBJU.2022.05.01

**Published:** 2022-08-03

**Affiliations:** 1 Universidade do Estado do Rio de Janeiro Unidade de Pesquisa Urogenital Rio de Janeiro RJ Brasil Unidade de Pesquisa Urogenital - Universidade do Estado do Rio de Janeiro - Uerj, Rio de Janeiro, RJ, Brasil; 2 Hospital Federal da Lagoa Rio de Janeiro RJ Brasil Serviço de Urologia, Hospital Federal da Lagoa, Rio de Janeiro, RJ, Brasil

The September-October number of Int Braz J Urol, the 18th under my supervision is special. The International Brazilian Journal of Urology new impact factor just been released and we have a spectacular result: 3.050. We are now on the same level as the traditional urology journals. In less than three years of our tenure as editor-in-chief, we have achieved through hard and serious work tripling the journal’s impact factor and making the journal one of the most important in the field in the world. We must celebrate this achievement and continue with our work. We are on the right track and we will surely increase even more our impact factor and our relevance in the academic environment.

In this number the Int Braz J Urol presents original contributions with a lot of interesting papers in different fields: Ureteral stones, male breast cancer, Fournier’s Gangrene, Reconstructive urology, Renal cancer, Bladder pain syndrome, Renal stones, Renal Cysts, Hypogonadism, Robotic Surgery, Prostate Cancer. The papers came from many different countries such as Brazil, India, Indonesia, China, Turkey and USA and as usual the editor’s comment highlights some of them. The editor in chief would like to highlight the following works:

Dr. Sharma and colleagues from India, presented in page 739 (1) a nice systematic review about the efficacy of alpha-blockers as medical expulsive therapy (MET) and concluded that among the three commonly used alpha-blockers silodosin is the most efficacious drug as MET for lower ureter stones followed by alfuzosin and tamsulosin.

Dr. Makdissi and colleagues from Brazil performed in page 760 (2) a interesting narrative review about the male breast cancer (MaBC) urological aspects and concluded that despite its rarity, MaBC represents an important problem in men’s health that can be neglected if professionals who have higher access to this population are uninformed. Therefore, urologists can play an important role in the early diagnosis of MaBC because their work involves a broader scenario in which the focus is greater than sexual dysfunction and screening for prostate cancer.

Dr. Raizandha and colleagues from Indonesia performed in page 771 (3) a interesting systematic review about the role of Hyperbaric oxygen therapy (HBOT) in management of Fournier’s Gangrene and concluded that the adjunctive therapy of Hyperbaric Oxygen possessed a significantly lower mortality rate compared to conventional therapy. However, the effect of HBOT on the length of stay and number of debridement was not proven in this study.

Dr. Cao and colleagues from China performed in page 784 (4) a nice study to confirm the hypothesis that a Nomogram can be built to predict the pathological T3a upstaging from clinical T1a in patients with localized renal cell carcinoma before surgery and concluded that older age, higher ratio of the tumor maximum and minimum diameter (ROD), increased fibrinogen level (FIB), and larger tumor size were independent risk factors for upstaging. The ARFS model (Age, ROD, FIB, tumor Size) has a high prediction efficiency for pT3a upstaging in patients with cT1a Renal cell carcinoma.

Dr. Hacad and colleagues from Brazil performed in page 807 (5) a interesting study about the effects of biofeedback (BF) and manual therapy (MT) associated with transcutaneous electrical nerve stimulation (TENS) or postural exercises (PE) in the treatment of bladder pain syndrome (BPS) in women regarding pain and urinary symptoms and concluded that biofeedback and manual therapy associated with postural exercises showed a significant improvement in perineal and suprapubic pain and urinary symptoms after treatment and during follow-up. Both results suggest a possible role for the use of this physiotherapy technique to treat BPS patients.

Dr. Sahan and colleagues from Turkey performed in page 817 (6) an important study about a novel nomogram and a simple scoring system for urinary leakage after percutaneous nephrolithotomy and concluded that the novel scoring system is easy to use and repeatable. The efficacy of the factors predicting urinary leakage in the scoring system was demonstrated to be in agreement with the literature. In addition, this scoring system can be used as a predictive method to determine which patients should receive a DJ catheter intra-operatively to shorten the length of hospital stay by estimating the risk of urinary leakage and to decrease additional anesthesia exposure due to postoperative DJ catheter requirement.

Dr. Caglayan and colleagues from Turkey performed in page 830 (7) a interesting study about a deep learning model in detecting kidney stones in different planes according to stone size on unenhanced computed tomography (CT) images and concluded that the use of deep learning algorithms for the detection of kidney stones is reliable and effective. Additionally, these algorithms can reduce the reporting time and cost of CT-dependent urolithiasis detection, leading to early diagnosis and management.

Drs. Meng and Mi from China performed in page 842 (8) a study about the clinical efficacy and safety of transurethral flexible ureteroscopic incision and drainage with holmium laser in the treatment of parapelvic renal cysts and concluded that transurethral flexible ureteroscopic incision and drainage with holmium laser in the treatment of parapelvic renal cysts has obvious advantages over traditional surgery, and is worthy of advancement and application, but its long-term effect needs further follow-up studies.

The Editor-in-chief expects everyone to enjoy reading.
